# Hypertension and orthostatic hypertension in 85-year-olds and associations with mortality and cognitive decline in a longitudinal cohort study

**DOI:** 10.1038/s41598-025-94913-2

**Published:** 2025-03-27

**Authors:** Simon Ståhl, Peder af Geijerstam, Magnus Wijkman, Maria M. Johansson, John Chalmers, Katarina Nägga, Karin Rådholm

**Affiliations:** 1 Department of Internal Medicine, Department of Health, Medicine and Caring Sciences, Linköping University, Norrköping, Sweden; 2 Primary Health Care Center Cityhälsan Centrum, and Department of Health, Medicine and Caring Sciences, Faculty of Medicine and Health Sciences, Linköping University, Linköping, Sweden; 3 Department of Activity and Health, Department of Health, Medicine and Caring Sciences, Linköping University, Linköping, Sweden; 4 Department of Acute Internal Medicine and Geriatrics, Department of Health, Medicine and Caring Sciences, Linköping University, Linköping, Sweden; 5 The George Institute for Global Health, University of New South Wales, Sydney, NSW Australia; 6 Primary Health Care Center Kärna, and Department of Health, Medicine and Caring Sciences, Linköping University, Linköping, Sverige

**Keywords:** Hypertension, Orthostatic hypertension, Elderly, Cognitive decline, Mortality., Circulation, Cardiology, Risk factors

## Abstract

Hypertension is a risk factor for cardiovascular disease, but the evidence for treatment and blood pressure (BP) targets in the elderly is less robust. Orthostatic hypertension is a potential risk factor for cardiovascular mortality and cognitive decline. All 85-years-olds in Linköping municipality, Sweden, were invited to a prospective birth cohort study including questionnaires, cognitive testing and physical examinations, including supine and orthostatic BP measurements. Logistic regression and Cox proportional hazard models were used to assess the impact of baseline supine and orthostatic BP on cognitive decline and all-cause mortality after 5- and 7.2 years respectively. Of 650 invited 85-year-olds, 322 were included. During follow-up, 190 persons died, and 28 persons developed cognitive decline. Neither elevated supine BP nor orthostatic hypertension were associated with cognitive decline. After adjustments, elevated supine BP was not associated with mortality in all participants, but was associated with lower all-cause mortality in participants with previously diagnosed hypertension, HR 0.59 (95% CI 0.41–0.85). Orthostatic hypertension was not associated with all-cause mortality, HR 0.56 (95% CI 0.26–1.22) after multiple adjustments. In 85-year-olds with known hypertension, elevated supine BP was associated with lower all-cause mortality. Orthostatic hypertension was not associated with cognitive decline but trended towards a lower all-cause mortality.

**Supplementary Information:**

The online version contains supplementary material available at 10.1038/s41598-025-94913-2.

## Introduction

Hypertension is a well-known and treatable risk factor for cardiovascular disease which affects more than a billion adults worldwide^1^. The incidence increases with age, and studies indicate that individuals above 80 years old also benefit from treatment^2,3^. However, randomized trials are few, and in the most elderly and frail, normal or lower systolic blood pressure (BP) and use of BP-lowering medications is associated with higher mortality and cognitive decline^4–6^.

Orthostatic hypertension (OHT) is defined as increased BP in response to standing, and is associated with all-cause mortality, cardiovascular mortality, cerebrovascular disease, and cognitive decline^7,8^. In 2023, it was included in the European Society of Hypertension guidelines as a “special hypertension phenotype” along with orthostatic hypotension^1^. However, studies in the oldest population are few and generalizability may be limited because of selected cohorts^9–11^.

Despite increased recognition of OHT, there is no uniform definition, which may partly explain the variation in prevalence figures in previous studies of 0.6 to 39%^9,10,12–16^. A commonly used definition of OHT is an increase in systolic BP ≥ 20 mmHg within 1 to 3 min of standing. However, studies vary in their use of systolic and/or diastolic BP measurements, BP cut-offs and timings of BP measurements^7,9–14,16–26^. To address the lack of a uniform definition, recently published consensus statements have proposed the nomenclatures exaggerated orthostatic pressor response (EOPR), or exaggerated BP response to standing (ERTS), defined as a supine to standing systolic BP increase ≥ 20 mmHg (for EOPR, persistent in all readings of the first 5 min of standing; for ERTS, an average of readings after 1, 2, and 3 min of standing); and OHT, defined as EOPR or ERTS with a mean standing systolic BP ≥ 140 mmHg (for EOPR, mean after 3 and 5 min of standing; for ERTS, not defined)^27,28^, Box 1. However, these proposed definitions and their association with adverse outcomes have not been sufficiently studied.

**Figure:**
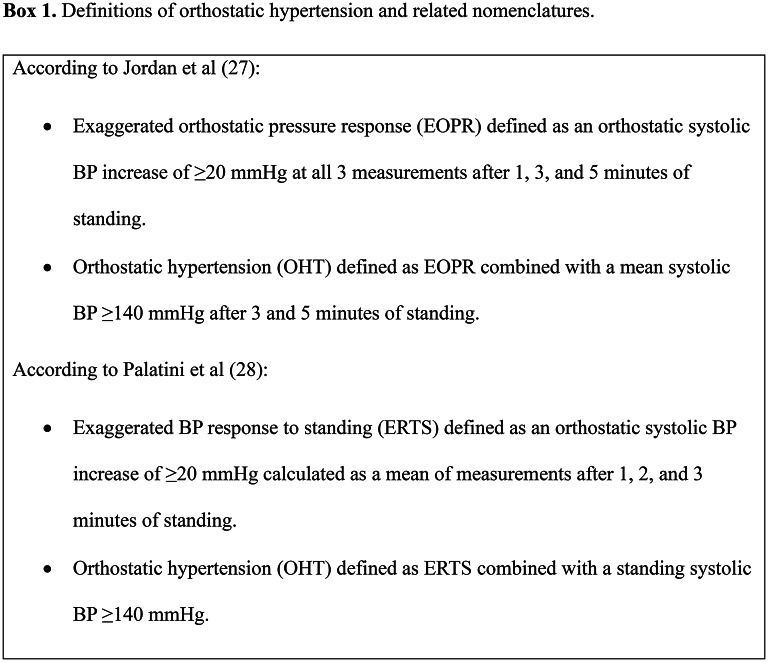

The purpose of the current study was to assess the prevalence of hypertension, EOPR and OHT according to different definitions in a Swedish population of 85-year-olds, as well as their association with cognitive decline and mortality at follow-up.

## Materials and methods

### Study population

Participants were from the population-based, longitudinal cohort study Elderly in Linköping Screening Assessment (ELSA-85). Details about data collection have been published before^29^. In summary, all inhabitants born in 1922 in the municipality of Linköping (*n* = 650), a middle-sized city in Sweden, were identified through the population register and invited by postal mail to participate in the baseline assessment between March 2007 and March 2008. Those who accepted participation answered questionnaires regarding their social status, physical and mental health, and medical history, and were assessed with cognitive and laboratory testing, as well as electrocardiography (ECG). Cognitive testing was repeated after 5 years and mortality was followed for a median of 7.2 years. In the present study, participants with valid supine and orthostatic BP measurements at baseline were included.

**Fig. 1:**
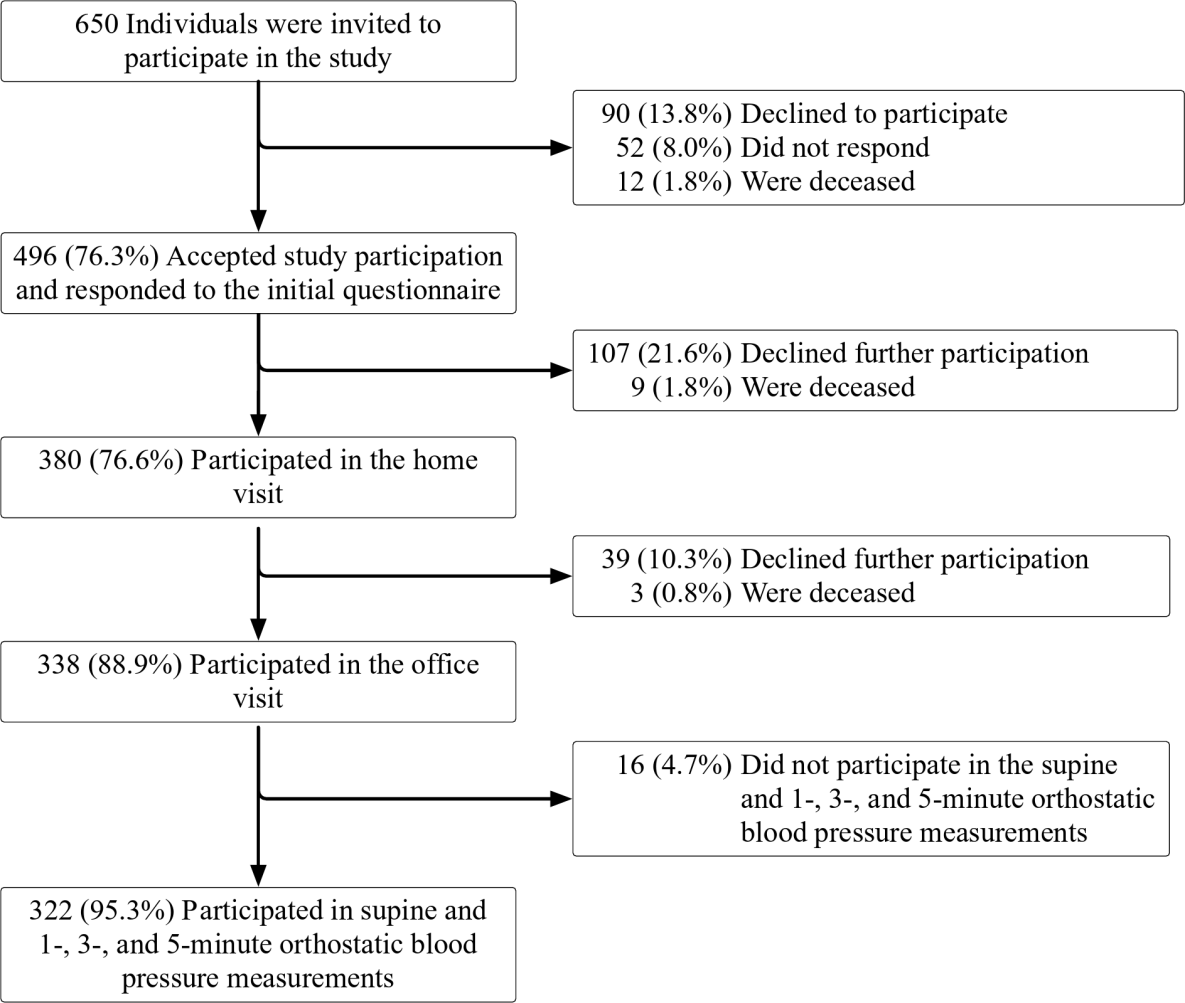
Flow chart for included participants. Percentages are of the preceding step in the flow chart.

At the 5-years follow-up, 113 completed the data collection, Fig. 1. Date of death was collected from medical charts and time to follow-up was calculated as the difference between the date of inclusion and date of death. Those who were not deceased during the study were censored at October 11th, 2015.

## Blood pressure measurements

At baseline, supine BP was recorded manually by trained medical staff after 5 minutes’ rest. To assess the impact of baseline BP on all-cause mortality and cognitive decline, participants were grouped based on supine BP and current definitions of hypertension as < 140/90 mmHg (< 140 mmHg systolic and < 90 mmHg diastolic) or ≥ 140/90 mmHg (≥ 140 mmHg systolic and/or ≥ 90 mmHg diastolic)^1^. Sub-group analyses were made based on baseline hypertension status, where those who reported a previous diagnosis of hypertension or current use of any BP-lowering medication (renin-angiotensin-aldosterone system [RAAS] inhibitors, calcium channel blockers, thiazide diuretics, betablockers or spironolactone) were classified as “known hypertension”, and all others were classified as “no known hypertension”. Beta blockers but not loop-diuretics were included as antihypertensives, as the baseline data was collected in 2007 when betablockers were still considered a first line antihypertensive treatment.

After supine BP measurements, participants were asked to stand up and standing BP was measured after 1, 3, 5 and 10 min. EOPR was defined as in the consensus statement by Jordan et al. as a supine to standing systolic BP increase ≥ 20 mmHg at 1, 3 and 5 min of standing^27^, and ERTS was defined as in the consensus statement by Palatini et al. as a supine to standing systolic BP increase ≥ 20 mmHg, calculated as a mean of the 1-, 3- and 5 min standing measurements^28^.

Orthostatic hypertension was defined as according to Jordan et al.^27^, as EOPR in combination with a mean of the 3- and 5-minute standing systolic BP ≥ 140 mmHg. Participants were excluded if any of the supine, 1-, 3-, or 5- minute systolic BP measurements were missing or if they had orthostatic hypotension, defined as a standing BP decrease of at least 20 mmHg systolic or 10 mmHg diastolic after 1, 3, 5 or 10 min^30^.

For exploratory analyses we applied wider definitions more akin to many previous studies: for EOPR/ERTS, a supine to standing systolic BP increase ≥ 20 mmHg after any of 1, 3, 5 or 10 min, and for OHT, EOPR/ERTS in combination with a mean of the 3- and 5-minute standing systolic BP ≥ 140 mmHg^11,31,32^.

### Other measurements

The Mini-Mental State Examination (MMSE) is a widely used screening tool for cognitive impairment with a high sensitivity and specificity for dementia^33^. It consists of 20 items assessing orientation, memory, attention, language, and visuospatial function, with a maximum score of 30 points^34^. We defined cognitive decline as either acquiring a dementia diagnosis during follow up and/or a decrease in MMSE score ≥ 3 points, which has previously been shown to represent a significant cognitive decline^35^. The diagnosis of dementia was retrieved from participants’ medical records. The ICD-10 criteria were employed for dementia diagnoses^36^. The diagnosis was established through a comprehensive evaluation that consisted of a thorough medical history taking, physical examination including neurological and psychiatric evaluation, cognitive tests and blood tests to rule out other potential causes of cognitive decline.

Information about previous or current diagnosis of heart failure, diabetes, stroke, neurological disease, and dementia was collected manually from medical records. Information about diagnosis of hyperlipidemia, hypertension, previous myocardial infarction, and current medication was obtained from self-reported questionnaires. Body mass index (BMI) was calculated by dividing weight (kg) by squared height (m). Thyroid stimulating hormone (TSH), N-terminal prohormone brain natriuretic peptide (NT-proBNP) and creatinine were analyzed from non-fasting venous blood samples. Serum creatinine, age and sex was used to calculate the estimated glomerular filtration rate (eGFR) using the 2021 Chronic Kidney Disease Epidemiology Collaboration (CKD-EPI)^37^. The presence of atrial fibrillation was assessed manually from a standard 12-lead ECG. Polypharmacy was defined as having 5 or more prescribed medications at baseline^38^.

### Statistical analyses

Statistical analyses were performed using IBM SPSS statistics 29.0.1.1, R version 4.4.1 and RStudio version 2024.04.2 + 764 (RStudio, Inc., Boston, MA, USA). A *P* value of < 0.05 was considered statistically significant for all analyses. Normality was evaluated using histogram plots and a Kolmogorov-Smirnov test. Continuous variables were reported as the mean and standard deviation (SD), or median and interquartile range (IQR) for those with a skewed distribution. Differences between groups were assessed using a *t*-test for normally distributed variables and a Mann-Whitney U-test for variables with a skewed distribution. Categorical variables were reported as numbers and percentages, and differences between groups were tested using a Chi-squared test, or a Fisher’s exact test when the cell count did not reach 5 in one or more cells.

Cox proportional hazard models were used to assess the associations between BP status, EOPR/ERTS and OHT status at baseline with all-cause mortality after a median 7.2-year follow-up, respectively. Models were unadjusted (Model 1), adjusted for sex (Model 2), and adjusted for sex, use of BP-lowering medication, statin medication, eGFR, cardiovascular and neurodegenerative comorbidities and type 2 diabetes (Model 3). For EOPR and OHT, supine systolic BP was also included in Model 3.

Similarly, logistic regression models were used to assess the associations between BP status, EOPR/ERTS and OHT status at baseline and cognitive decline at the 5-year follow-up. Models were unadjusted (Model 1), adjusted for sex (Model 2) and adjusted for sex, use of BP-lowering medications, statin medication, eGFR, cardiovascular comorbidities and type 2 diabetes (Model 3). For EOPR and OHT, supine systolic BP was also included in Model 3.

### Ethical considerations

Ethical approval for the ELSA-85 study was given by the Research Ethics Committee of Linköping University, Sweden (Dnr 141-06, 332 − 31, 455 − 31) and the study adhered to the Declaration of Helsinki. Informed consent was obtained from all the participants before enrollment in the study.

## Results

Of 650 85-year-olds invited to participate, 338 (52.0%) participated in the baseline office visits, and 322 (95.3%) of those had valid supine and 1-, 3-, and 5-minute orthostatic BP measurements and were thus included in this study. The remaining either declined participation, did not respond, died before complete data collection, or were excluded due to incomplete or invalid data, Fig. 1. Of 322 included individuals, 186 (57.8%) were women and 237 (73.6%) had elevated supine BP at baseline. Of participants, 241 (74.8%) reported one or more cardiovascular comorbidities.

**Table 1:** Baseline characteristics of all participants depending on supine blood pressure status, and orthostatic reaction status.

		All, *N* = 322	Supine blood pressure status			Orthostatic reaction status ^a^		
			< 140/90 mmHg, *n* = 85	≥ 140/90 mmHg, *n* = 237	*P*	No OHT, *n* = 130	OHT, *n* = 22	*P*
Women, n (%)		186 (57.8)	47 (55.3)	139 (58.6)	0.591	84 (64.6)	10 (45.5)	0.087
BMI (kg/m^2^), median (Q1-Q3)		26.0 (23.0–28.0)	25.0 (23.0–28.0)	26.0 (23.0–28.0)	0.724	26.0 (23.0–28.0)	25.5 (22.8–27.3)	0.586
eGFR (mL/min/1.73 m^2^), median (Q1-Q3)		58.7 (48.2–71.8)	57.9 (45.4–69.9)	59.3 (49.1–73.1)	0.179	53.3 (45.6–70.5)	68.5 (63.3–80.4)	< 0.001
TSH (mIE/L), median (Q1-Q3)		1.5 (1.0–2.4)	1.4 (0.8–2.3)	1.6 (1.1–2.5)	0.170	1.5 (1.0–2.2)	1.7 (0.9–2.8)	0.519
NT-proBNP (ng/L), median (Q1-Q3)		380.0 (210.0–860.0)	460.0 (210.0–1275.0)	360.0 (210.0–770.0)	0.144	375 (200.0–930.0)	265.0 (157.5–752.5)	0.239
Living in nursing home, n (%)		4 (1.2)	1 (1.2)	3 (1.3)	> 0.99	3 (2.3)	0	> 0.99
Ever-smoker, n (%)		77 (23.9)	21 (24.7)	56 (23.6)	0.842	25 (19.2)	6 (27.3)	0.397
Comorbidities, n (%)								
Atrial fibrillation		48 (14.9)	20 (23.5)	28 (11.8)	0.007	21 (16.2)	3 (13.6)	> 0.99
Hypertension		175 (54.3)	34 (40.0)	141 (59.5)	0.002	71 (54.6)	9 (40.9)	0.234
Heart failure		54 (16.8)	26 (30.6)	28 (11.8)	< 0.001	32 (24.6)	1 (4.5)	0.047
Previous cardiovascular events		108 (33.5)	40 (47.1)	68 (28.7)	0.002	44 (33.8)	5 (22.7)	0.302
Diabetes		58 (18.0)	14 (16.5)	44 (18.6)	0.666	21 (16.2)	2 (9.1)	0.531
Neurological disease or dementia		21 (6.5)	4 (4.7)	17 (7.2)	0.429	6 (4.6)	1 (4.5)	> 0.99
Any cardiovascular disease ^b^		241 (74.8)	66 (77.6)	175 (73.8)	0.560	101 (77.7)	12 (54.5)	0.022
BP-lowering medications, n (%)								
Any BP-lowering medication ^c^		218 (67.7)	65 (76.6)	153 (64.6)	0.049	91 (70.0)	9 (40.9)	0.007
RAAS inhibitors		106 (32.9)	32 (37.6)	74 (31.2)	0.290	50 (38.5)	2 (9.1)	0.007
Calcium channel blockers		57 (17.7)	15 (17.6)	42 (17.7)	0.975	28 (21.5)	2 (9.1)	0.249
Beta blockers		134 (41.6)	44 (51.8)	90 (38.0)	0.029	57 (43.8)	4 (18.2)	0.022
Thiazide diuretics		49 (15.2)	11 (12.9)	38 (16.0)	0.487	17 (13.1)	4 (18.2)	0.512
Spironolactone		16 (5.0)	7 (8.2)	9 (3.8)	0.143	9 (6.9)	1 (4.5)	> 0.99
Loop diuretics		73 (22.7)	29 (34.1)	44 (18.6)	0.004	40 (30.8)	1 (4.5)	0.010
Other medications, n (%)								
Nitrates		53 (16.5)	17 (20.0)	36 (15.2)	0.312	17 (13.1)	1 (4.5)	0.474
Antidepressants		35 (10.9)	8 (9.4)	27 (11.4)	0.607	12 (9.2)	3 (13.6)	0.460
Sedatives		59 (18.3)	16 (18.8)	43 (18.1)	0.902	27 (20.8)	4 (18.2)	> 0.99
Statins		84 (26.1)	27 (31.8)	57 (24.1)	0.171	34 (26.2)	0	0.004
Polypharmacy, n (%) ^d^		176 (54.7)	56 (65.9)	120 (50.6)	0.017	73 (56.2)	9 (40.9)	0.172
MMSE, median (Q1-Q3)		28.0 (26.0–29.0)	28.0 (26.0–29.0)	28.0 (27.0–29.0)	0.595	28.0 (27.0–29.0)	28.5 (27.0–29.0)	0.609
BP measurements, mean (SD)								
Supine systolic		151.6 (21.6)	125.6 (8.6)	161.0 (16.7)	NA	147.1 (22.6)	154.5 (23.4)	0.176
Supine diastolic		73.6 (10.4)	65.5 (7.9)	76.5 (9.7)	NA	70.4 (10.4)	77.0 (10.7)	0.012
Pulse pressure, median (Q1-Q3)		75.0 (65.0–90.0)	60.0 (55.0–65.0)	80.0 (73.5–95.0)	< 0.001	76.7 (18.9)	75.0 (60.0–100.0)	0.990
Orthostatic measurements, mean (SD)								
1 min	Systolic	140.9 (24.3)	119.1 (16.5)	148.7 (21.8)	< 0.001	144.9 (23.0)	165.3 (19.2)	< 0.001
	Diastolic	74.2 (13.6)	66.2 (9.4)	77.0 (13.7)	< 0.001	73.9 (10.5)	83.0 (12.7)	0.004
3 min	Systolic	142.3 (24.1)	121.3 (15.4)	149.8 (22.1)	< 0.001	146.3 (22.4)	169.2 (21.3)	< 0.001
	Diastolic	74.1 (11.5)	66.0 (9.8)	77.0 (10.6)	< 0.001	73.8 (10.4)	82.9 (14.1)	0.008
5 min	Systolic	142.1 (23.7)	122.1 (15.5)	149.3 (22.0)	< 0.001	145.9 (22.5)	169.7 (22.1)	< 0.001
	Diastolic	74.4 (11.8)	66.8 (9.5)	77.1 (11.3)	< 0.001	74.1 (10.7)	83.2 (12.5)	0.003
10 min	Systolic	144.6 (24.5)	125.1 (15.9)	151.6 (23.2)	< 0.001	147.5 (22.6)	174.6 (25.4)	< 0.001
	Diastolic	75.3 (11.5)	68.5 (9.7)	77.8 (11.2)	< 0.001	75.1 (11.1)	82.7 (13.1)	0.014

a. Participants with orthostatic hypotension excluded. b. Including previous myocardial infarction, previous stroke or transient ischemic attack within the last six months. c. Including RAAS-inhibitors, calcium channel blockers, thiazide diuretics, beta blockers and spironolactone.d. Five or more prescribed drugs. Abbreviations: OHT, orthostatic hypertension; BMI, body mass index; eGFR, estimated glomerular filtration rate; TSH, thyroid stimulating hormone; NT-proBNP, N-terminal pro B-type natriuretic peptide; BP, blood pressure; RAAS, renin-angiotensine-aldosterone system; MMSE, mini mental state examination.

**Table 2:** Odds ratios and 95% CI for the composite outcome of cognitive decline after 5 years in individuals without dementia at baseline with supine blood pressure ≥ 140/90 vs. < 140/90 MmHg, presented for all participants, as well as participants with and without known hypertension. Also presented are the odds ratios for the composite outcome for participants with OHT vs. no OHT, and EOPR vs. no EOPR, for all participants without dementia at baseline and orthostatic hypotension.

		Cognitive decline at the 5-year follow-up		
		**Model 1**	**Model 2 (sex)**	**Model 3 (multiple)** ^**a**^
BP ≥ 140/90 vs. < 140/90 mmHg at baseline (ref)		**OR (95% CI)**	**OR (95% CI)**	**OR (95% CI)**
All participants, *n* = 309	1 (ref)	0.69 (0.28–1.74)	0.69 (0.27–1.74)	0.69 (0.26–1.83)
Known hypertension ^b^, *n* = 224	1 (ref)	0.66 (0.22–1.99)	0.62 (0.20–1.91)	0.53 (0.15–1.90)
Not known hypertension, *n* = 85	1 (ref)	0.78 (0.15–4.00)	0.81 (0.15–4.23)	1.26 (0.17–9.27)
OHT (*n* = 21) vs. not OHT (ref, *n* = 126)				
Participants without orthostatic hypotension, *n* = 147	1 (ref)	0.74 (0.13–4.12)	0.98 (0.16–6.05)	1.22 (0.16–9.58)
EOPR (*n* = 24) vs. not EOPR (ref, *n* = 123)				
All participants without orthostatic hypotension, *n* = 147	1 (ref)	0.61 (0.11–3.33)	0.89 (0.15–5.40)	1.04 (0.15–7.40)

a. Model 3 was adjusted for sex, use of blood pressure-lowering medication, statin medication, estimated glomerular filtration rate, cardiovascular disease and diabetes type 2. For analyses of OHT and EOPR supine systolic blood pressure was also included.

b. A previous hypertension diagnosis or use of one or more anti-hypertensive drugs.

Abbreviations: BP, blood pressure; EOPR, exaggerated orthostatic pressor response; OHT, orthostatic hypertension.

Differences between groups were tested using Chi square test, or Fishers exact test when cell count did not reach 5 or more in one cell, for categorical variables, a two sampled t-test for normally distributed continuous variables, and a Mann-Whitney U test for continuous variables with a skewed distribution.

### Baseline characteristics by hypertension status

Of all participants, 233 (72.4%) reported either known hypertension diagnosis or use of at least one BP-lowering medication. Of participants with supine BP ≥ 140/90 vs. < 140/90 mmHg, known hypertension diagnosis was reported by 141 (59.5%) vs. 34 (40.0%), *P =* .002, use of at least one BP-lowering medication was reported by 153 (64.6%) vs. 65 (76.6%), *P* = .049, and polypharmacy was reported by 120 (50.6%) vs. 56 (65.9%), *P* = .017, Table 1.

In participants with known vs. not known hypertension, median eGFR was 67.7 (Q1-Q3 53.9–77.2) vs. 54.8 (Q1-Q3 45.6–69.4), *P* < .001, median BMI was 26.0 (Q1-Q3 23.6–29.0) vs. 25.0 (Q1-Q3 23.0–26.0), *P* < .001, atrial fibrillation was present in 46 (19.7%) vs. 2 (2.2%), *P* < .001, heart failure was reported by 52 (22.3%) vs. 2 (2.2%), *P* < .001, previous cardiovascular events was reported by 92 (39.5%) vs. 16 (18.0%), *P* < .001, and diabetes was reported by 53 (22.7) vs. 5 (5.6%) participants, *P* < .001, Supplementary Table 1.

### Baseline characteristics by orthostatic hypertension status

Orthostatic hypertension, EOPR and ERTS according to consensus criteria were present in the same 3 individuals (0.9%), when using the criteria by Jordan et al. and Palatini et al., respectively, and due to the small number of individuals, outcome analyses were not performed for these group. Of participants with valid orthostatic measurements at 1, 3, 5, and 10 min (*n* = 312), using the wider OHT and EOPR/ERTS definitions (henceforth referred to as OHT and EOPR, respectively), 22 (7.1%) had OHT and 130 (41.7%) had a normal orthostatic reaction. The remaining 160 (51.3%) had orthostatic hypotension (*n* = 154) or both orthostatic hypotension and OHT (*n* = 6) (due to different changes in BP for diastolic vs. systolic BP, and/or at different time points of orthostatic measurements), and these were not included in the subsequent analyses. Comparing individuals with OHT vs. no OHT, use of BP-lowering medications was reported by 9 (40.9%) vs. 91 (70.0%), *P* = .007, cardiovascular disease was reported by 12 (54.5%) vs. 101 (77.7%), *P* = .022, and median eGFR was 68.5 (Q1-Q3 63.3–80.4) vs. 53.3 (Q1-Q3 45.6–70.5) mL/min/1.73 m^2^, *P* < .001, Table 1. Using the same wider definition and again excluding participants with orthostatic hypotension, EOPR was present in 25 (8.0%) of participants (of which 22 also had OHT), Supplementary Table 1.

### Supine blood pressure, orthostatic hypertension and incident cognitive decline

Of the 322 included participants, 309 did not have dementia diagnosis at baseline. At follow-up after 5 years, 28 (9.1%) of these had declined in MMSE score with at least 3 points, and 6 (1.9%) had also developed dementia. Neither of supine BP ≥ 140/90 mmHg, OHT nor EOPR at baseline were associated with development of cognitive decline, Table 2.

### Supine Blood Pressure, Orthostatic Hypertension and Mortality

**Fig. 2:**
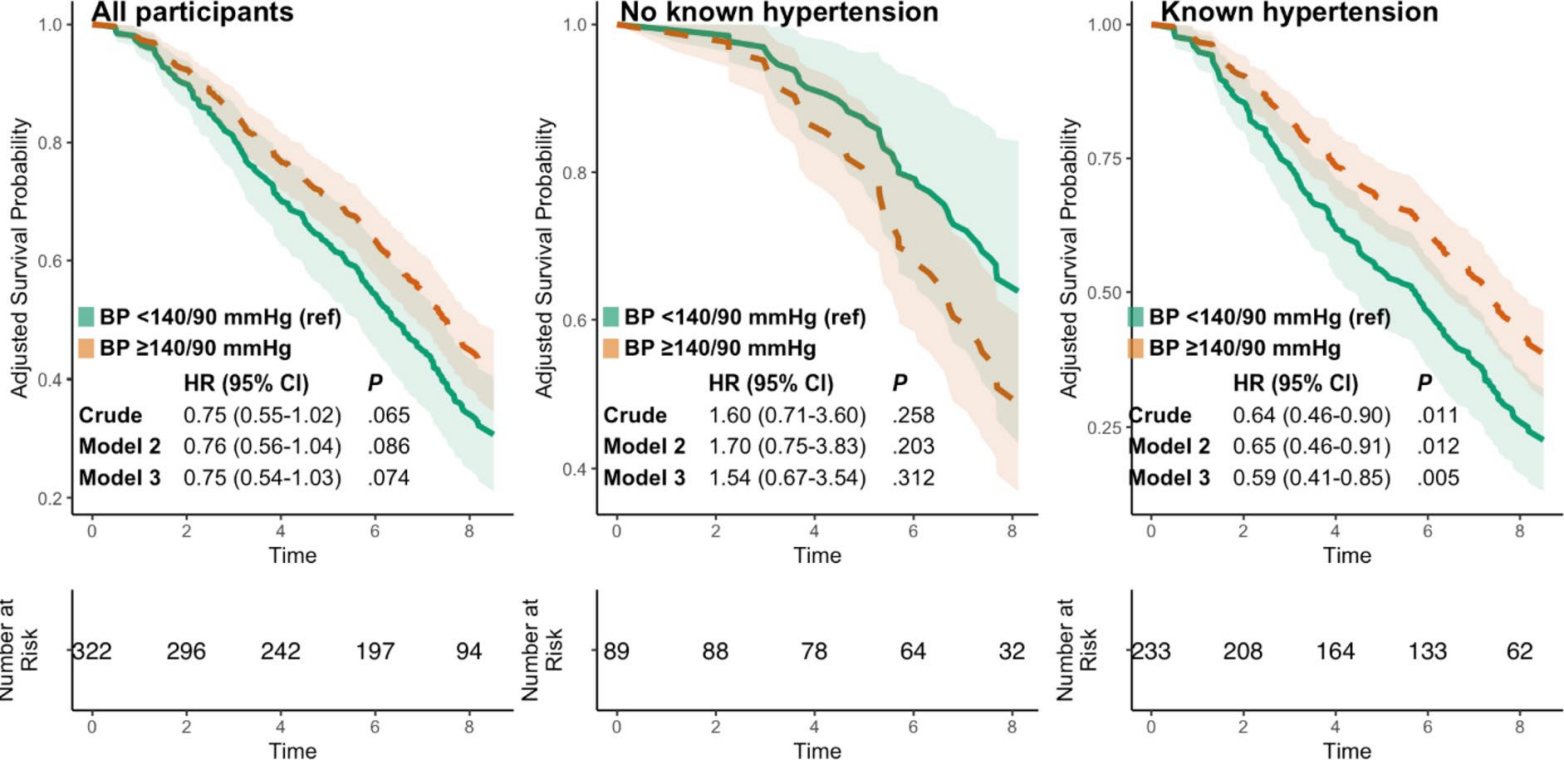
Survival probability for participants depending on office supine blood pressure status at baseline until a median of 7.2 years follow up.

**Fig. 3:**
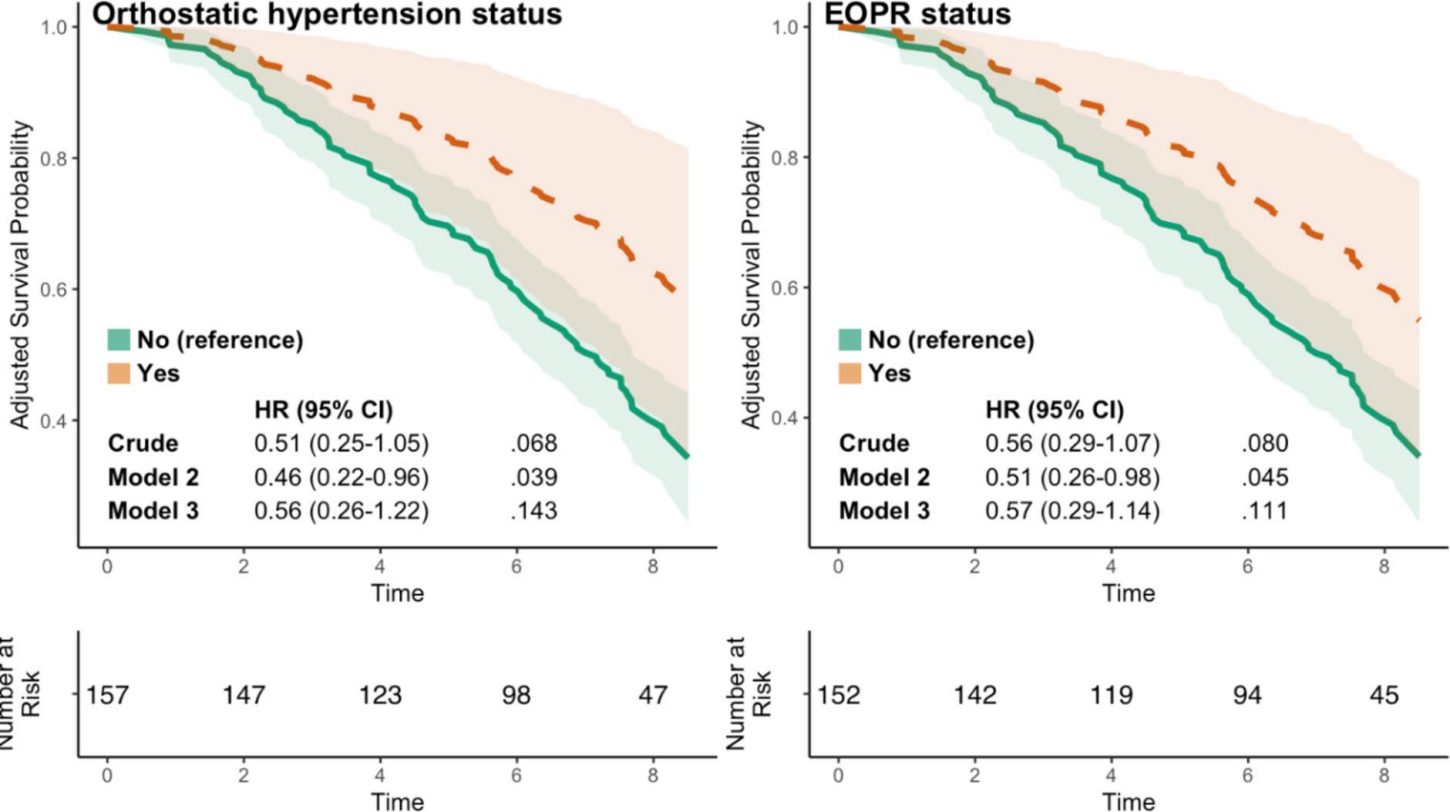
Survival probability for participants depending on orthostatic hypertension and exaggerated orthostatic pressure response status until a median of 7.2 years follow up.

Of the 322 included participants, 190 (59%) had died after a median follow-up of 7.2 years. Supine BP ≥ 140/90 mmHg at baseline was not significantly associated with mortality in all participants, HR 0.75 (95% CI 0.54–1.03) after a median of 7.2 years follow up, but was associated with lower all-cause mortality in participants with known hypertension, HR 0.59 (95% CI 0.41–0.85) for the fully adjusted model, Fig. 2. For participants with OHT vs. not OHT and EOPR vs. not EOPR, mortality was lower after adjusting for sex after a median of 7.2 years follow up, HR 0.46 (95% CI 0.22–0.96) and 0.51 (95% CI 0.26–0.98), respectively, but this association was no longer significant after multiple adjustments, Fig. 3.

## Discussion

In this study of 322 85-year-olds, OHT was present in 0.9% of participants when defined using the consensus criteria^27^, and 7.1% of participants when using the wider OHT definition. In participants with known hypertension, elevated BP at baseline was associated with lower all-cause mortality after 7.2 years. Neither supine BP ≥ 140/90 mmHg, OHT, nor EOPR at baseline were associated with cognitive decline, but orthostatic hypertension trended towards lower all-cause mortality.

### Prevalence of orthostatic hypertension

Prevalence of OHT according to the consensus definitions was lower than in most previous studies, where prevalence varied between 0.6% and 39%, depending on the studied cohort and the definition of OHT^7,9–17,21,39^. However, a recent study on hypertensive adults with a mean age of 33 years found OHT in 0.74% of participants, defining OHT as a systolic BP increase to standing of ≥ 20 mmHg and a standing systolic BP ≥ 140 mmHg^16^. However, given the much younger population, one would expect a higher prevalence in our population since previous studies suggest that OHT may be an effect of increased vascular resistance^40^. Variations in OHT-prevalence may thus depend on cohort demographics, but also, and perhaps more significantly, differences in the definition of OHT. In studies of populations more akin to our, OHT was more prevalent. In a general population of 1004 85-year-olds, 4% had OHT defined as a systolic BP increase ≥ 20 mmHg from sitting to one minute of standing^10^. This was comparable to the 6.8% we found using the wider definition of OHT. Other studies from similar age groups found a higher prevalence. A study of 530 geriatric patients with a mean age of 83 years found a 22.3% prevalence^31^, and a study of nursing home residents with a mean age of 88 years found a 28% prevalence^9^. These two studies, however, may have included a cohort with more co-morbidities than ours. Finally, adverse events have been reported with a supine to standing systolic BP increase of 6.5 mmHg, which may imply that a lower cut-off could be more appropriate^25^.

### Orthostatic hypertension and associations with All-Cause mortality

In our sex-adjusted model, we found a lower risk of death among those with OHT compared to normal orthostatic reaction, indicating that sex may affect this association. However, this was attenuated in the multiple adjusted model. In our sample, participants with OHT reported both less cardiovascular co-morbidities and less use of BP-lowering medications, indicating that this sub-sample had a better cardiovascular health at baseline compared to the group with a normal orthostatic reaction, which may have contributed to the lower unadjusted mortality risk in this group. Several studies have found associations between OHT and adverse outcomes, including all-cause mortality and cardiovascular mortality^8,12,39^. However, in a large Swedish cohort including 32 669 participants, OHT was associated with a lower risk of hospitalization due to heart failure^23^. Another study on individuals with diabetes found that diastolic orthostatic hypertension was associated with a lower risk of cardiovascular events^21^.

Our results were similar for the EOPR and OHT group, which may be explained by the large overlap between the two groups. Almost all previous research has analyzed OHT looking only at the orthostatic reaction (i.e., EOPR), thus the impact of absolute standing BP values remains unclear. Future studies, with larger study samples, are needed to better assess this.

### Baseline blood pressure and association with All-Cause mortality

Among participants with known hypertension, we found that the risk of death until follow up was reduced if baseline BP was elevated. This is in line with previous observational studies, where a higher BP in elderly with BP-lowering treatment was associated with a lower mortality rate^4^. Similarly, studies have seen increased risk of mortality among elderly and frail populations with low or normal BP^6,41^, especially if systolic BP was below 130 mmHg. In our study, participants with known hypertension had higher BMI, worse renal function, and more often reported diabetes and other cardiovascular diseases (apart from hypertension) than those with not know hypertension. Possibly, a low BP is more detrimental to health in these elderly individuals due to the risk of, for example falls and other adverse effects of a low BP. However, in contrast to these results, large studies, including randomized controlled trials, have found protective effects of BP-lowering treatment among the most elderly, including elderly frail individuals^2,3^. Thus, the increased risk seen among participants with supine BP < 140/90 mmHg might reflect reverse causation. For example, low BP could be a result of poor fluid and food intake among persons approaching the end of life, in which case the mortality rate increases due to factors other than the BP-level^6^.

### Baseline blood pressure and orthostatic hypertension, associations with cognitive decline

We found no significant association between baseline supine BP, OHT or EOPR and cognitive decline. This contrasts with some previous research which found associations between OHT and cognitive decline as well as lacunar strokes^7,42^. However, the participants in our study had a high MMSE score at baseline, with a median value of 28.0 points (Q1-Q3 26.0–29.0), indicative of a cognitively fit population. Furthermore, it is possible that 85-years-olds with cognitive impairment at baseline or follow up were more likely to decline to participate or drop out during the study. Therefore, a possible association might have been missed.

### Strengths and limitations

Several methodological aspects should be considered. Approximately half of the invited cohort was not included in the baseline analyses, with non-participants more frequently being nursing home residents, suggesting that the included sample represents a relatively healthier population. As expected in a cohort of 85-year-olds, attrition due to death was substantial during follow-up, increasing the risk of bias and necessitating cautious interpretation of the results. Furthermore, only 2 cases of cognitive decline were observed in individuals with EOPR during follow-up, limiting the interpretability of the findings. Thus, the study may be underpowered, reducing its ability to draw conclusions for associations with mortality or cognitive decline. In addition, as an observational study, despite adjusting for several important confounders, no conclusions regarding causality can be drawn.

Despite multiple recent efforts to produce uniform criteria for OHT, the varied criteria´s from previous studies complicates comparison between studies. Supine and orthostatic BP were only measured on one occasion, whereas repeated recordings are needed for diagnosis of hypertension, and recommended for diagnosis of OHT^27^. In fact, in a study where orthostatic measurements were taken 3 times during a 3.5-month period, no participant met the criteria for OHT at all measurements^16^, questioning the generalizability from a single measurement. In our study resting BP was measured in the supine position, whereas a sitting to standing BP measurement is more suitable to perform in a clinical setting, and most frequently used for measurement of resting BP. A supine to standing change in posture will trigger a greater physiological response^43^ and is also proposed in the consensus documents. Additional research is needed to determine whether a sitting to standing orthostatic reaction has similar effects as the supine to standing.

However, the study is unique in including the most elderly part of the population with a long observation time and serves as a novel contribution to the ongoing research of OHT. Additional research is needed to further assess if specific BP targets, or more specified indications for BP-lowering treatment are needed for the most elderly population.

In summary, for elderly with known hypertension, elevated office BP was associated with lower all-cause mortality. Orthostatic hypertension was not associated with cognitive decline but trended towards a lower all-cause mortality. Prevalence of OHT and EOPR/ERTS varied greatly depending on the definition that was used, but were very low when using the consensus definitions. Further research is needed to better understand whether OHT is a condition worth clinical consideration in this age group.

## Electronic supplementary material

Below is the link to the electronic supplementary material.

Supplementary Material 1

Supplementary Material 2

Supplementary Material 3

## Data Availability

The dataset supporting the conclusions of this article are available from authors on reasonable request.

## Abbreviations

BMI: body mass index
BP: blood pressure
eGFR: estimated glomerular filtration rate
EOPR: exaggerated orthostatic pressor response
MMSE: Mini Mental State Examination
NT-proBNP: N-terminal pro B-type natriuretic peptide
OHT: orthostatic hypertension
RAAS: renin-angiotensin-aldosterone system
TSH: thyroid stimulating hormone

## Declarations

### Data sharing statement

The dataset supporting the conclusions of this article are available from authors on reasonable request.
