# Supplementary material for: Hypertension and orthostatic hypertension in 85-year-olds and associations with mortality and cognitive decline in a longitudinal cohort study
Source: Sci Rep. 2025 Mar 27;15:10529. doi: 10.1038/s41598-025-94913-2 (PMC11950220; doi:10.1038/s41598-025-94913-2)
Supplement: Supplementary file 1 — Supplementary Material 1 [file 41598_2025_94913_MOESM1_ESM.pdf]

Hypertension and Orthostatic Hypertension in 85-year-olds: Associations Over Time With Mortality and Cognitive Decline

Authors (surnames underlined): Simon Ståhl (a), Peder af Geijerstam (b), Magnus Wijkman (a), Maria M. Johansson (c, d), John Chalmers (e), Katarina Nägga (d), Karin Rådholm* (b, e)

Supplementary table 1. Baseline characteristics of all participants depending on previous hypertension status and EOPR-status.

Variables	All, N = 322	History of hypertension		*P*	Orthostatic reaction [a]		*P*
		Known hypertension, n = 233	No known hypertension, n = 89		No EOPR or OH, n = 127	EOPR, n = 25	
**Women, n (%)**	186 (57.8)	142 (60.9)	44 (49.4)	.062	82 (64.6)	12 (48.0)	.119
**BMI (kg/m^2), median (Q1-Q3)**	26.0 (23.0-28.0)	26.0 (23.6-29.0)	25.0 (23.0-26.0)	<.001	26.0 (23.0-28.0)	25.0 (23.0-27.0)	.413
**eGFR (mL/min/1.73m^2), median (Q1-Q3)**	58.7 (48.2-71.8)	67.7 (53.9-77.2)	54.8 (45.6-69.4)	<.001	53.3 (45.7-70.6)	68.5 (62.7-75.5)	.002
**TSH (mIE/L), median (Q1-Q3)**	1.5 (1.0-2.4)	1.4 (1.0-2.3)	1.5 (1.0-2.5)	.658	1.5 (1.0-2.2)	1.4 (0.7-2.7)	.986
**NT-proBNP (ng/L), median (Q1-Q3)**	380.0 (210.0-860.0)	250.0 (140.0-485.0)	460.0 (240.0-1080.0)	<.001	370.0 (200.0-895.0)	310.0 (160.0-855.0)	.482
**Living in nursing home, n (%)**	4 (1.2)	3 (1.3)	1 (1.1)	<.99	3 (2.4)	0	>.99
**Ever-smoker, n (%)**	77 (23.9)	50 (21.5)	27 (30.3)	.095	25 (19.7)	6 (24.0)	.625
**Comorbidities, n (%)**							
Atrial fibrillation (on ECG)	48 (14.9)	46 (19.7)	2 (2.2)	<.001	21 (16.5)	3 (12.0)	.766
Hypertension	175 (54.3)	52 (22.3)	0	<.001	69 (54.3)	11 (44.0)	.344
Heart failure	54 (16.8)	52 (22.3)	2 (2.2)	<.001	30 (23.6)	3 (12.0)	.198
Previous cardiovascular events	109 (33.9)	92 (39.5)	16 (18.0)	<.001	43 (33.9)	6 (24.0)	.335
Diabetes	58 (18.0)	53 (22.7)	5 (5.6)	<.001	21 (16.5)	2 (8.0)	.371
Neurological disease or dementia	21 (6.5)	14 (6.0)	7 (7.9)	.546	6 (4.7)	1 (4.5)	>.99
**Cardiovascular composite [b]**	241 (74.8)	222 (95.3)	19 (21.3)	<.001	98 (77.2)	15 (60.0)	.072
**BP-lowering medications, n (%)**							
Any BP-lowering medication [c]	218 (67.7)	218 (93.6)	0	<.001	88 (69.3)	12 (48.0)	.035
RAAS inhibitors	106 (32.9)	106 (45.5)	0	<.001	49 (38.6)	3 (12.0)	.010
Calcium channel blockers	57 (17.7)	57 (24.5)	0	<.001	27 (21.3)	3 (12.0)	.412
Beta blockers	134 (41.6)	134 (57.5)	0	<.001	55 (43.3)	6 (24.0)	.067
Thiazide diuretics	49 (15.2)	49 (21.0)	0	<.001	16 (12.6)	5 (20.0)	.347
Spironolactone	16 (5.0)	16 (6.9)	0	.008	9 (7.1)	1 (4.0)	>.99
Loop diuretics	73 (22.7)	69 (29.6)	4 (4.5)	<.001	39 (30.7)	2 (8.0)	.018
**Other medications, n (%)**							
Nitrates	53 (16.5)	46 (19.7)	7 (7.9)	.011	17 (13.4)	1 (4.0)	.310
Antidepressants	35 (10.9)	23 (9.9)	12 (13.5)	.334	11 (8.7)	4 (16.0)	.276
Sedatives	59 (18.3)	47 (20.2)	12 (13.5)	.177	26 (20.5)	5 (20.0)	.943
Statins	84 (26.1)	75 (32.2)	9 (10.1)	<.001	32 (25.2)	2 (8.0)	.057
**Polypharmacy, n (%) [d]**	176 (54.7)	161 (69.1)	15 (16.9)	<.001	70 (55.1)	12 (48.0)	.488
**MMSE, median (Q1-Q3)**	28.0 (26.0-29.0)	28.0 (26.0-29.0)	27.4 (27.0-29.0)	.366	28.0 (27.0-29.0)	29.0 (27.0-29.0)	.662
**Blood pressure measurements, mean (SD)**							
Supine systolic	151.6 (21.6)	151.1 (22.5)	153.0 (19.4)	.439	147.7 (22.4)	150.4 (24.8)	.621
Supine diastolic	73.6 (10.4)	73.0 (10.8)	75.2 (9.4)	.078	70.5 (10.4)	75.4 (11.2)	.055
**Pulse pressure, median (Q1-Q3)**	75.0 (65.0-90.0)	66.3 (11.7)	69.1 (11.1)	.974	75.0 (65.0-90.0)	70.0 (60.0-97.5)	.400

**Orthostatic measurements, mean (SD)**								
**1 min**	Systolic	145.1 (23.2)	140.1 (25.0)	143.1 (22.3)	.323	145.4 (23.1)	160.6 (22.2)	.004
	Diastolic	73.9 (10.6)	73.4 (14.0)	76.4 (12.3)	.058	73.9 (10.6)	81.8 (12.4)	.005
**3 min**	Systolic	146.6 (22.9)	141.4 (24.3)	144.8 (23.4)	.245	146.7 (22.5)	164.5 (23.8)	.002
	Diastolic	74.1 (10.6)	73.0 (11.4)	76.9 (11.4)	.008	73.9 (10.5)	81.4 (13.9)	.017
**5 min**	Systolic	146.1 (22.7)	140.9 (23.8)	145.3 (23.2)	.132	146.4 (22.5)	164.5 (25.3)	.002
	Diastolic	74.4 (10.9)	73.3 (11.7)	77.3 (23.2)	.007	74.2 (10.8)	81.6 (12.5)	.009
**10 min**	Systolic	147.5 (22.5)	143.4 (24.3)	147.7 (24.7)	.164	147.5 (22.8)	171.0 (25.8)	<.001
	Diastolic	75.1 (11.1)	74.4 (11.5)	77.8 (11.3)	.018	75.1 (11.2)	81.9 (12.6)	.017

a. Participants with orthostatic hypotension excluded.

b. Including previous myocardial infarction, previous stroke or transient ischemic attack within the last 6 months.

c. Including RAAS-inhibitors, calcium channel blockers, thiazide diuretics, beta blockers and spironolactone.

d. Five or more prescribed drugs.

Differences between groups were tested using Chi square test, or Fishers exact test when cell count did not reach 5 or more in one cell, for categorical variables, a two sampled t-test for normally distributed continuous variables, and a Mann-Whitney U test for continuous variables with a skewed distribution.

Abbreviations: BMI, body mass index; BP, blood pressure; eGFR, estimated glomerular filtration rate; EOPR, exaggerated orthostatic pressor response; MMSE, Mini Mental State Examination; NT-proBNP, N-terminal pro B-type natriuretic peptide; OHT, orthostatic hypertension; RAAS, renin-angiotensin-aldosterone system; TSH, thyroid stimulating hormone.
