# Supplementary material for: Hypertension and orthostatic hypertension in 85-year-olds and associations with mortality and cognitive decline in a longitudinal cohort study
Source: Sci Rep. 2025 Mar 27;15:10529. doi: 10.1038/s41598-025-94913-2 (PMC11950220; doi:10.1038/s41598-025-94913-2)
Supplement: Supplementary file 2 — Supplementary Material 2 [file 41598_2025_94913_MOESM2_ESM.pdf]

Hypertension and Orthostatic Hypertension in 85-year-olds: Associations Over Time With Mortality and Cognitive Decline

Authors (surnames underlined): Simon Ståhl (a), Peder af Geijerstam (b), Magnus Wijkman (a), Maria M. Johansson (c, d), John Chalmers (e), Katarina Nägga (d), Karin Rådholm* (b, e)

Supplementary figure 1. A Direct Acyclic Graph (DAG) used to evaluate adjustments for mortality during follow-up.

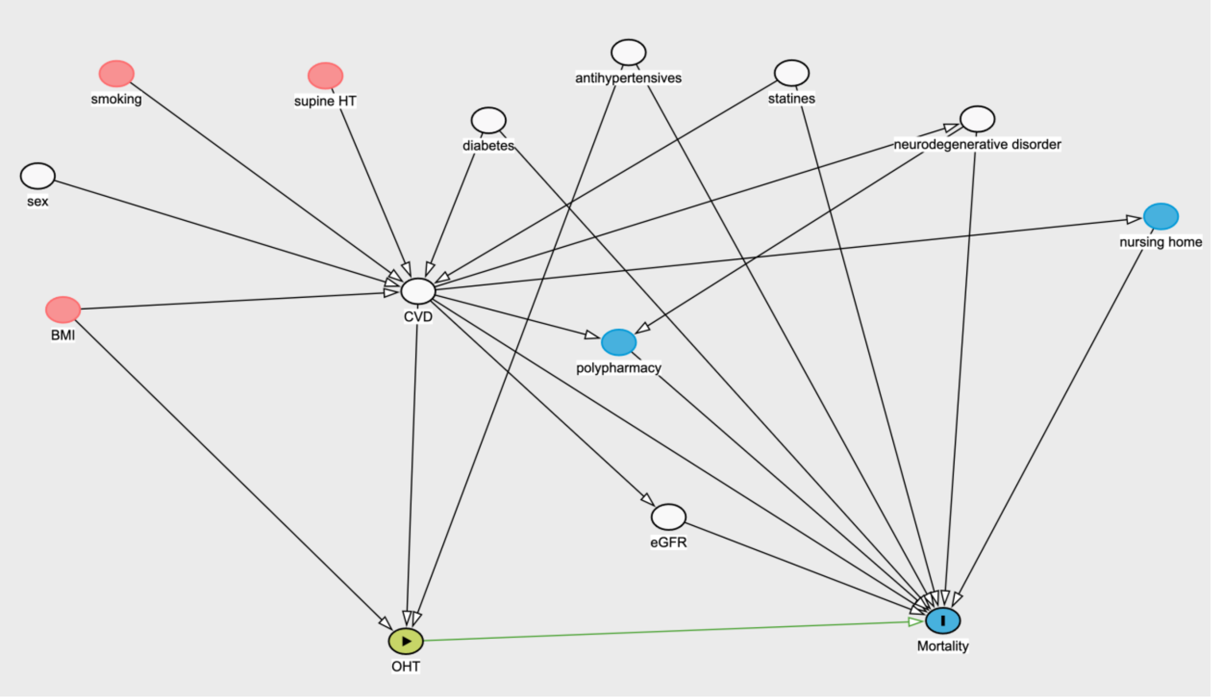

Exposure, Outcome, Unadjusted variable, Adjusted variable, Causal path, Biasing path
