# Supplementary figures and images for: Hypertension and orthostatic hypertension in 85-year-olds and associations with mortality and cognitive decline in a longitudinal cohort study
Source: Sci Rep. 2025 Mar 27;15:10529. doi: 10.1038/s41598-025-94913-2 (PMC11950220; doi:10.1038/s41598-025-94913-2)

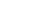 Exposure, 
 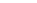 Outcome, 
 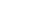 Unadjusted variable, 
 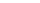 Adjusted variable, 
 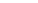 Causal path, 
 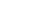 Biasing path

Supplement: Supplementary file 3 — Supplementary Material 3 [file 41598_2025_94913_MOESM3_ESM.pdf]
